# Sentence Recall in Latent and Anomic Aphasia: An Exploratory Study of Semantics and Syntax

**DOI:** 10.3390/brainsci11020230

**Published:** 2021-02-12

**Authors:** Christos Salis, Nadine Martin, Laura Reinert

**Affiliations:** 1 Speech & Language Sciences, Newcastle University, Newcastle upon Tyne NE1 7RU, UK; 2 Communication Sciences and Disorders, Eleanor M. Saffran Center for Cognitive Neuroscience, Temple University, Philadephia, PA 19122, USA; nmartin@temple.edu (N.M.); reinertlauram@gmail.com (L.R.)

**Keywords:** sentence recall, semantic plausibility, syntax, aphasia, latent aphasia, anomic aphasia

## Abstract

We investigated whether semantic plausibility and syntactic complexity affect immediate sentence recall in people with latent and anomic aphasia. To date, these factors have not been explored in these types of aphasia. As with previous studies of sentence recall, we measured accuracy of verbatim recall and uniquely real-time speech measures. The results showed that accuracy did not distinguish performance between latent aphasia and neurotypical controls. However, some of the real-time speech measures distinguished performance between people with latent aphasia and neurotypical controls. There was some evidence, though not pervasive, that semantic plausibility and syntactic complexity influenced recall performance. There were no interactions between semantic plausibility and syntactic complexity. The speed of preparation of responses was slower in latent aphasia than controls; it was also slower in anomic aphasia than both latent and control groups. It appears that processing speed as indexed by temporal speech measures may be differentially compromised in latent and anomic aphasia. However, semantic plausibility and syntactic complexity did not show clear patterns of performance among the groups. Notwithstanding the absence of interactions, we advance an explanation based on conceptual short-term memory as to why semantically implausible sentences are typically more erroneous and possibly also slower in recall.

## 1. Introduction

Immediate spoken sentence recall (or sentence recall) is a simple yet discerning measure of verbal abilities (language and short-term memory, STM), which engages simultaneously multiple linguistic representations, phonological, semantic and conceptual [1,2,3,4,5]. Clinically, it is used in diagnostic aphasia assessments—for example, Comprehensive Aphasia Test [6] and Western Aphasia Battery [7]—as well as in aphasia treatment [8,9]. The present exploratory study involved secondary data analyses and examined sentence recall in latent and anomic aphasia. Unlike anomic aphasia, latent aphasia is not a well-defined or well-studied type of aphasia. In the literature, different diagnostic adjectives which qualify the term “aphasia” or “dysphasia” have been used, such as minimal dysphasia [10], latent dysphasia [11,12], subclinical aphasia [13]. Spoken naming ability, a cardinal diagnostic feature of aphasia, as measured by confrontation naming tests, is within normal limits in latent aphasia [12,14]. Latent aphasia is usually undetected by standard aphasia tests [12,15,16,17]. However, some authors have subsumed it under anomic aphasia [18,19,20], indirectly acknowledging that language abilities in people with latent aphasia are somewhat mildly impaired. Furthermore, recent evidence suggests that despite the within-normal-limits performance in the aphasia test, communication difficulties are reported by such individuals [21]. Similar to other researchers [16], in this study, we define latent aphasia as a diagnostic entity above the cut-off point of the Western Aphasia Battery [7]. We focus on two linguistic factors, semantic plausibility and syntactic complexity, which have been dominant themes in the sentence processing literature in aphasia, recall and comprehension. Both factors interact with other domains of cognition, notably STM and inhibitory control; yet, to date, they have not been studied in latent and anomic aphasia, although syntactic processing (comprehension and production) in anomic aphasia has received some attention [22,23,24]. Our study is unique because it used temporal speech measures in addition to standard accuracy analysis. Temporal speech measures are real-time measures that examine durations of silent and speech segments, thus affording greater precision, which helps to refine our understanding of the underlying cognitive processes that underpin recall [25,26]. Such measures can differentiate people with latent from those with anomic aphasia and neurotypical controls [15,16,27]. We should note that the present study is exploratory and utilized opportunistic, secondary data with a small number of experimental stimuli. Despite this insurmountable limitation, which renders our findings provisional, our objective is to broaden the theoretical debate of sentence recall abilities in latent and anomic aphasia and discuss the diagnostic potential of our methods. 

### 1.1. Semantic Plausibility

Evidence for the role of semantic plausibility in sentence recall (and sentence processing more broadly) comes from studies that juxtapose implausible (e.g., # worms eat birds) and plausible sentences (e.g., birds eat worms). Unlike plausible sentences, implausible ones violate a person’s real-life experience and presumably semantic knowledge [28,29,30]. For example, birds eat worms, but worms do not eat birds. Although such linguistic contrasts have attracted attention in literature domains beyond aphasia [31,32,33], they remain largely unexplored in aphasia. Newcombe and Marshall [34] investigated sentence recall in people with focal, left hemisphere lesions, with and without aphasia. The authors described the latter subgroup as showing “residual dysphasic symptoms” [34] (p. 329), a description which may align with the phenotype of latent aphasia. In comparison to controls, both groups made more errors in implausible than plausible sentences. There were no differences between people with or without aphasia. Using diverse sentence structures, other authors found that implausible sentences are recalled less accurately than plausible sentences [35,36,37,38]. This line of enquiry of mainly case studies has shown that implausible sentences diminish recall accuracy in people with moderate and possibly latent aphasia. The important question is why implausible sentences are challenging.

In aphasia and other corners of the literature, researchers have appealed to the construct of inhibitory control and its interactions with semantic knowledge and syntax (discussed in Section 1.2) to explain the plausibility effect in sentence recall. Inhibitory control (also known as interference or attentional control) is ascribed to the suppression of irrelevant or interfering information for optimizing and ultimately achieving a particular goal [39,40]. Typically, inhibitory control measures such as the ubiquitous Stroop [41] contrast two conditions. One condition induces interference between a required response (i.e., goal) and a stimulus, which must be inhibited in order to achieve the required goal as dictated by the task. The other condition serves as a baseline, neutral condition of minimal interference. A difference in the two conditions reveals inhibitory control costs and consequences, be it in terms of lower accuracy and/or slower response time. Accordingly, plausible and implausible sentences resemble the two main conditions of inhibitory control measures. In the context of sentence recall, meaningful and familiar information, which is activated in plausible sentences, is thought to be more resistant to interference than less meaningful information when recalling implausible sentences [32]. This happens presumably because the novel conceptual event triggered by a plausible sentence is congruent with semantic representations in long-term memory (LTM). In inhibitory control terms, plausible sentences mitigate inhibitory control demands, whereas implausible sentences elevate them. In the aphasia literature, related explanations have featured—for example, difficulty in suppressing, inhibiting or reconciling conflict [34,36,38]. Such explanations resonate with others from sentence comprehension studies in ageing [42,43,44]. Researchers suggested that, unlike younger adults, older adults may have difficulty inhibiting knowledge activated by implausible sentences. In turn, this gives rise to increased comprehension errors and protracted reaction times in implausible sentences in comparison with plausible ones. Furthermore, plausibility may interact with syntactic complexity, such that performance in implausible and syntactically complex sentences may be worse than in simpler implausible sentences. In the present study, we explore these issues.

### 1.2. Syntactic Complexity and Semantic Plausibility

Syntactic complexity is an indelible theme in aphasia research. In general, reversible declarative sentences with simple syntax (2 a, 2 b) tend to be understood and produced more accurately than sentences with complex syntax (2 c) [45,46,47,48].

(2 a) the boy _(Agent)_ is kissing the girl _(Patient)_
(2 b) the girl _(Agent)_ is kissing the boy _(Patient)_
(2 c) the girl _(Patient)_ is being kissed by the boy _(Agent)_

In such sentences and depending on the verb and its selectional restrictions in terms of animacy/non-animacy, the thematic roles expressed by the nouns can occupy the syntactic position of either Agent or Patient, resulting in an equally plausible event semantically, if swapped [29,49]. Moreover, sentences (2 a) and (2 b) follow the canonical—that is, frequent—thematic role order in English of Agent–Patient, whereas in (2 c), the order of thematic roles is Patient–Agent and therefore non-canonical and infrequent. Contrastively, semantically non-reversible sentences (examples below) often comprise animate and inanimate nouns where the Agent, if inanimate, can occupy only one syntactic position for the sentence to be semantically plausible (3 a, 3 c). Otherwise, the sentence is implausible (3 b, 3 d).

(3 a) the girl _(Agent)_ is kicking the ball _(Patient)_
(3 b) # the ball _(Agent)_ is kicking the girl _(Patient)_
(3 c) the ball _(Patient)_ is being kicked by the girl _(Agent)_
(3 d) # the girl _(Patient)_ is being kicked by the ball _(Agent)_

Plausibility and its possible interaction with syntax has been studied very little in sentence recall in aphasia. The person reported by Friedrich and colleagues [36] was able to repeat non-reversible sentences well (96% correct) but more complex sentences were less successful (54% correct). Newcombe and Marshall’s findings [34] are less clear-cut. However, the research theme of reversibility, semantic plausibility and syntactic complexity has attracted more attention in sentence comprehension studies in aphasia. Older research [50,51] that examined comprehension of sentences with simple syntax found that implausible sentences such as (3 b) are problematic in aphasia, although people with seemingly latent aphasia performed as well as neurotypical controls [52]. More recent research has failed to find statistically reliable effects of plausibility in sentences with simple and complex syntax [19,53]. Furthermore, older research suggests that implausible sentences with complex syntax may also be problematic for some people with aphasia [54,55].

### 1.3. Motivation of the Present Study

Sentence recall engages momentarily diverse linguistic representations through their activation from LTM, akin to depth of processing [2,56,57]. In memory research, this activation and subsequent processing is achieved through inhibitory control as well as processing speed [56,58,59,60], which may also exist in sentence recall [61]. Inhibitory control is deficient in some people with aphasia [62,63,64,65,66]. As discussed earlier, implausible sentences are thought to increase inhibitory control demands and result in poorer recall than plausible sentences in people with aphasia. However, it is unclear whether aphasia severity affects recall of implausible sentences. To date, only Newcombe and Marshall [34], to our knowledge, explored this issue; they did not find a difference between the two groups of people with left hemisphere damage whom they studied. Previous findings [12,15,16] suggest that language skills, both in terms of accuracy as well as speed of delivery, are affected in latent aphasia. Influenced by processing speed theory [60], DeDe and Salis [15] proposed that latent aphasia is characterized by a faulty simultaneity mechanism—that is, an inability to simultaneously plan and execute multiple linguistic and other cognitive operations [60]. The effect of syntactic complexity on sentence recall, and especially of implausible sentences, is another unexplored issue in mild forms of aphasia. While syntactic complexity (in plausible sentences) often has a negative effect on sentence processing, its effect on sentence recall and whether aphasia severity affects recall is largely unknown.

Recent studies in neurotypical individuals and people with aphasia have shown that inhibitory control may be crucial in facilitating processing of complex sentences [67,68,69]. We mentioned earlier that simple sentences follow the expected or canonical order of thematic roles, whereas non-canonical sentences do not. Canonicity of thematic role order in two-argument, declarative sentences is the way we define syntactic complexity in the present study (see Section 2). If inhibitory control is deficient in aphasia, this may explain, at least in part, why complex sentences are difficult for many people with aphasia. When it comes to syntactic complexity and plausibility, an implausible sentence would add to the syntactic complexity demand. Consequently, recall of implausible complex sentences will be less accurate and slower than plausible simple sentences. People with latent aphasia will be more accurate and faster than people with anomic aphasia but less accurate and slower than neurotypical controls.

Apart from clinical reasons [70], knowing about sentence recall abilities in people with milder forms of aphasia could help us to understand the relationship of STM and LTM in these individuals. The interactive relationship of these constructs has been debated for decades in mainstream cognitive psychology [56,57,58,71] as well as aphasiology [36,72,73]. Gradually, these debates have strengthened arguments for the importance of inhibitory or attentional control mechanisms in regulating activation of representations in LTM [58,59,74,75]. In the language research domain, memory and attentional control are thought to contribute to language abilities (comprehension and production) in neurotypical individuals [64,67,76,77,78].

Along similar reasoning, Potter and Lombardi [3,4,5,79] proposed a model of sentence recall which emphasizes the importance of the conceptual representation of the event triggered by the sentence, which, upon recall, is regenerated (that is, reconstructed) from the message level, rather than just from the phonological representation. An explanatory mechanism in Potter’s model is its discrete STM component that stores the conceptual representation of the sentence. This sets it apart from other models of STM that assume interactions with LTM solely through attentional control [56,58]. The conceptual component interacts with LTM and is needed to process novel stimuli and interpretations not yet stored in LTM. Nevertheless, upon recall, sentence regeneration stems from LTM, which carries, in its course of production, syntactic as well as conceptual semantic and syntactic representations, which have been activated in LTM [5,79]. On the basis of this theoretical view, these mechanisms may be differentially impaired in latent and anomic aphasia, which we will explore in Section 4.

## 2. Materials and Methods

### 2.1. Participants

Individuals with latent (*n* = 17) and anomic (*n* = 27) aphasia were selected from AphasiaBank [80]. The 93.8 cut-off Aphasia Quotient figure of the Western Aphasia Battery (WAB-Revised) [7] was used to differentiate diagnostic group membership and aphasia severity. Inclusion criteria were: English as primary language and absence of a motor speech disorder (apraxia of speech, dysarthria), if reported. Two participants were reported to have depression (acwt04a—latent group; williamson08a—anomic group). Three participants had suffered a previous stroke (scale16a, wozniak06a—latent group; adler08a—anomic group). We excluded individuals with reported neurological conditions (e.g., seizures, meningitis, apraxia of vision) and hearing problems. The included participants and their respective AphasiaBank codes are shown in Appendix A. Because sentence recall is included in the standard protocol of AphasiaBank, we were able to find and utilize data from only one neurotypical person (mscuc2b) who had completed the sentence repetition task. Additional data from neurotypical controls were collected from volunteer participants in the USA (*n* = 8) and four native speakers of American English in the UK.

### 2.2. Materials

The data elicitation task was the Repetition Test, IIB Sentence task of the AphasiaBank protocol, which comprises 12 sentences in total. For each participant, the data were generated originated from eight sentences in total (see Appendix B): four experimental sentences with semantic errors (i.e., implausible) and control sentences without errors (i.e., plausible). Based on canonicity, within each plausibility category, there were two simple (i.e., canonical) and two complex sentences (i.e., non-canonical). Thus, for each participant, eight responses were analyzed, with two trials per condition. The remaining four sentences of the task were presented to the participants and acted as fillers but were not analyzed. Apart from two participants in the latent aphasia group (tcu10b, tucson06b) and one in the anomic group (williamson09b), data from the first administration of the task were used. In these three participants, we could not locate the first administration of the task in AphasiaBank.

Demographic information, language and STM abilities (word span task of the AphasiaBank protocol) of the three groups are shown in Table 1. The three groups were similar in age, K-W (2) = 0.04, *p* = 0.9, years of education, K-W (2) = 0.74, *p* = 0.7. However, the distribution of sexes was different, χ^2^ (2) = 11.28, *p* < 0.01. The two clinical groups did not differ in time post onset, Wilcoxon two sample Z = 578, *p* = 0.48 (two tailed), but differed in the Aphasia Quotient, Wilcoxon two sample Z = 378, *p* < 0.001 (two tailed). In terms of word span, the three groups differed, K-W (2) = 24.29, *p* < 0.001. Dunn’s pairwise tests with Holm adjustment for multiple comparisons showed that: (i) the latent group differed significantly from the anomic group, *z* = −2.08, *p* < 0.05 [latent > anomic]; (ii) the anomic group differed from controls, *z* = −4.19, *p* < 0.001 [anomic < controls]; and (iii) the latent aphasia group differed from controls, z = −4.91, *p* < 0.01 [latent < controls].

Mean number of words in implausible sentences was 6.5 and in plausible sentences was 7.3. This difference was not significant, Wilcoxon two sample *z* = 0.5, *p* = 0.62 (two tailed). There was also no difference in the number of content vs. function words, Fisher exact, *p* = 0.28 (two tailed). With regard to syntax, there was no difference in number of words between simple (mean = 6.25) and complex sentences (mean = 7.75), Wilcoxon two sample *z* = 1.36, *p* = 0.17 (two tailed). Content and function words were equally distributed by syntactic complexity, Fisher exact, *p* = 0.41 (two tailed).

### 2.3. Data Elicitation and Dependent Measures

Audio files of the Repetition Test IIB were extracted from AphasiaBank videos and imported into Praat speech analysis software [81], which generated an acoustic spectrograph of each recording of the sentence recall task. With its semi-automated function of silent pause identification (see Appendix C), Praat segmented the spectrograph between speech and silent segments (see Appendix D). The minimum silence detection threshold was set at 200 ms [15,26,82,83] (shown in the first screenshot of Appendix C). A trained student assistant checked and adjusted manually as appropriate all segments generated by Praat for accuracy and also transcribed orthographically participant responses. Transcriptions included all words that were recalled accurately, all errors, filled pauses (e.g., “uh”, “erh”) and mazes that were not part of the stimuli (e.g., “I think”). The first author checked all Praat segmentations and orthographic transcriptions for accuracy.

We elicited five dependent measures, one based on accuracy of responses and four temporal measures: (1) Accuracy was measured with a Levenshtein distance in words algorithm [84], used previously in studies of sentence recall [85,86]. The algorithm counted the minimum number of errors (word substitutions, omissions, additions) between a stimulus sentence and a response. Error additions also included mazes and filled pauses as these speech behaviors are associated with linguistic processing difficulties [15,83,87]. The Levenshtein distance in words algorithm is a deviation from the target in single units, whereby zero reflects an errorless response and increasing units reflect greater number of errors within a response. The algorithm has good construct validity [85]. The stimuli and responses were transcribed orthographically in Microsoft Excel. The algorithm itself was implemented as a macro. Examples as to how the algorithm works with the stimulus sentence the dog chased the cat up the tree are as follows: (i) “the cat chased the dog up the tree” (2 errors); (ii) “the dog chases the {cat} uhm {up} tree” (4 errors). Because responses that are 100% correct have values of 0 in the Levenshtein distance metric, all responses were inverted by subtracting the number of errors from 12. Thus, a response with 2 errors (12 minus 2) was converted to 10, a response with 4 errors was converted to 8 and so on. (2) Response time was the total duration of a response, from the end of the experimenter’s stimulus presentation and its ensuing silent interval before recall onset to the very end of a participant’s spoken response. (3) Preparation time was the silent interval between the end of the examiner’s spoken stimulus sentence and the participants’ spoken response. (4) Speech time was the duration of the speech segments, excluding preparation time and any silent pauses within. (5) Pause time was the duration of all silent pauses within a response. When a response did not contain any pauses as defined in this study, a pause of zero ms was logged.

### 2.4. Statistical Analyses

Descriptive statistics (means and standard deviations) for each participant and for each sentence type were first generated, which were subsequently aggregated separately into the three groups. The main analysis was conducted in R [88] using the following packages: “base” (version 3.6.1), “emmeans” (version 1.4) and “multcomp” (version 1.4-13). For each of the five dependent measures, we conducted ANOVAs, using group and sentence type as main effects. The distribution of sexes in the groups differed (see Section 3) but this variable was not used as a covariate because, in preliminary analyses, it was not a significant factor. Additionally, we investigated two-way interactions between group and sentence type. For significant effects, we also carried out post hoc tests with Sidak corrections for multiple comparisons. Although the measures deviated from normality, we did not transform them, nor did we replace extreme value because such practices are not universally acceptable [89,90]. However, to improve the reliability of our analyses, we used permutations tests for linear models (“lmPerm” package). Post hoc pairwise comparisons were excluded from these analyses. The dataset has been submitted for transparency.

## 3. Results

Descriptive statistics (mean and SD) from accuracy and temporal speech analyses are shown in Table 2. In the accuracy measure, the higher the figure, the higher the accuracy; recall that these are the inverted values. Conversely, in all other measures, the higher the figure, the slower the response.

Accuracy: There was a main effect of group, F (2) = 22.933, *p* < 0.001. Post hoc tests showed no difference between control and latent groups (*p* = 0.16), the anomic group was less accurate than controls (*p* = 0.00), and the anomic was less accurate than the latent group (*p* = 0.00). There was also a main effect of sentence type, F (3) = 5.417, *p* = 0.0013. The results of pairwise comparisons showed that only the plausible complex–plausible simple contrast was significant (plausible complex > plausible simple). The interaction between group and sentence type was not significant, F (6) = 1.156, *p* = 0.33. Permutation tests generated the same results.

Response: There was a main effect of group, F (2) = 15.723, *p* < 0.001. Post hoc tests showed the controls were faster than the latent group (*p* = 0.01) and anomic groups (*p* = 0.00), and the latent group was faster than controls (*p* = 0.02). There was no main effect of sentence type, F (3) = 2.204, *p* = 0.09. The interaction between group and sentence type was not significant, F (6) = 0.462, *p* = 0.83. Permutation tests generated the same results. 

Preparation: There was a main effect of group, F (2) = 5.942, *p* = 0.003. Post hoc tests showed that the latent group was slower than controls (*p* = 0.017), the anomic group was slower than controls (*p* = 0.003), but no difference between latent and anomic groups (*p* = 0.94). There was also a main effect of sentence type, F (3) = 6.384, *p* < 0.001. The results of post hoc tests showed that preparation time for plausible simple sentences was faster than implausible simple (*p* = 0.016). It was also faster for plausible complex sentences in comparison to implausible simple (*p* = 0.018). No other comparison was significant. The interaction between group and sentence type was not significant, F (6) = 1.907, *p* = 0.08. Permutation tests generated the same results.

Speech: There was a main effect of group, F (2) = 10.150, *p* < 0.001. Post hoc tests did not reveal a difference between control and latent groups (*p* = 0.073); the controls were faster than the anomic group (*p* < 0.001); there was no difference between latent and anomic groups (*p* = 0.067). There was no main effect of sentence type, F (3) = 2.256, *p* = 0.229. There was no group by sentence type interaction, F (6) = 0.156, *p* = 0.98. Permutation tests generated the same results.

Pause: There was a main effect of group, F (2) = 14.070, *p* < 0.001. Post hoc tests showed no significant difference between control and latent groups (*p* = 0.063); the anomic group paused for longer than controls (*p* < 0.001) and longer than the latent group (*p* = 0.009). There was no main effect of sentence type, F (3) = 1.450, *p* = 0.229. Finally, there was no group by sentence interaction, F (6) = 0.252, *p* = 0.958. Permutation tests generated the same results.

## 4. Discussion

The main aim of this exploratory study was to improve our understanding of verbal abilities in latent and anomic aphasia, which, to date, has received scant attention. Our objective was to broaden the theoretical debate on sentence recall abilities in latent and anomic aphasia and discuss the diagnostic potential of our methods. We used accuracy as well as temporal speech measures and focused on two key factors, semantic plausibility and syntactic complexity, both of which are historical and topical [52,53]. We found several between-group differences in all five measures. Differences between sentence types were evident only in accuracy and preparation time. There were no group by sentence type interactions in any measure. Below, we summarize the key findings according to the main effects and discuss them in the context of previous research, mindful of the small number of stimuli (two per condition) in each sentence type, which is the main limitation of our study.

Ignoring the effect of sentence type, the accuracy measure did not distinguish performance between latent aphasia and controls. Accuracy was, however, discriminatory between latent and anomic as well as anomic and control groups. The lower accuracy of the anomic group in comparison to the other two groups could be attributed to word finding difficulties, the main feature of this type of aphasia. Similarly, the longer pauses observed in the anomic group in comparison to the other two groups could indicate lexical retrieval difficulties. In the other temporal speech measures, total duration of responses revealed that the latent aphasia group was slower than controls. Preparation of responses was slower in the latent aphasia group than controls, although pause and sentence duration did not distinguish these two groups. The anomic group differed from controls in all four measures, with controls outperforming the anomic group both in errors as well as speed (i.e., faster). The comparisons between the two clinical groups showed that the latent aphasia group outperformed the anomic group in terms of accuracy (greater in latent than anomic), total duration of response as well as the preparatory interval (latent faster in both measures than anomic). Considered together, these findings show that temporal speech measures may be diagnostically more discerning in revealing subtle or latent language problems after stroke, corroborating earlier findings [15,16,27]. That accuracy was similar between the latent group and controls but preparation of response was slower in the latent group than controls could be interpreted as a processing speed deficit [15,27]. It could also be that the latent aphasia group was more cautious in formulating their responses, which may have resulted in more protracted preparation time. Subjective difficulties in terms of processing speed after stroke in people who do not present with a diagnosis of aphasia have been reported in the literature [91]. In order to test these two competing hypotheses, use of self-reported processing speed measures could be used in addition to objective measures from experimental tasks. Accuracy is the standard way of investigating sentence recall. While past research [32,38] showed that it is discriminatory in relatively more severe forms of mild aphasia, such as anomic, accuracy as a measure on its own may not distinguish between latent aphasia and neurotypical controls. This finding highlights the importance of temporal speech measures in revealing subtle language deficits not only as a result of stroke [16,27] but also other neurological conditions, non-progressive and progressive [83].

Putting individual group performance aside, the main effect of sentence type showed that accuracy was higher in plausible simple than plausible complex sentences. This syntactic effect is consistent with previous findings from aphasia and neurotypical literature of sentence recall [29,45,62]. However, the lack of a semantic plausibility effect in the accuracy measure—that is, no difference between plausible and implausible sentences—deviates from past studies of sentence recall, which involved more severe forms of aphasia, e.g., conduction and transcortical sensory [36,37], than the two types we focused on in this study. It is possible, therefore, that accuracy as a measure of sentence recall may be less sensitive in milder than more severe forms of aphasia. It could also be that the linguistic stimuli in this study may not have been sensitive enough to elicit a semantic plausibility effect. The latter explanation seems unlikely because similar sentences in terms of number of words and syntactic complexity have been used previously in the neurotypical and aphasia literature [31,32,37]. Our findings also differ from Newcombe and Marshall [34], the only other study of sentence recall in possibly latent aphasia that we are aware of. More generally, inconclusive findings have been reported in sentence comprehension studies in aphasia, recent and older ones [19,53,54], which typically used sentence to picture matching or enactment [19]. Differences in the linguistic structure of the stimuli as well as data elicitation methods may account for the inconclusive findings in the literature.

Besides accuracy, preparation time was the only other measure that elicited a main sentence type effect. Preparation time in plausible simple sentences was faster than implausible simple sentences. It was also faster in plausible complex than implausible simple sentences. These findings could be interpreted as semantic plausibility effects. We did not find a syntactic complexity effect since preparation time was similar in the plausible simple–plausible complex and implausible simple–implausible complex contrasts. Furthermore, the absence of similar effects in other contrasts in preparation time (plausible simple–implausible complex; plausible complex–implausible complex) do not support our prediction, namely that implausible sentences would add to the syntactic complexity demand and result in slower preparation time.

To date, the semantic plausibility effect in sentence recall (both neurotypical and aphasia literature) has predominantly been conceived as a difficulty, inhibiting the plausible semantic representations stored in LTM that unavoidably semantically implausible representations temporarily activate [33,37,38]. This explanation appears congruent with the preparation time results that we obtained. Moreover, the absence of similar effects in the other temporal speech measures that we studied (response time, pause time, speech time) could be regarded as evidence that inhibitory control occurs at the stage of response formulation (or planning) as opposed to execution, since no sentence type effects were found in speech and pause time. While the role of inhibitory control on recalling implausible sentences has been debated in the literature, it is unclear at what level (or levels) of linguistic representation (that is, phonological, semantic, conceptual) it occurs. It is likely that different people with aphasia with differential processing difficulties at these levels, which are also known to affect STM abilities [68,73], may also have different underlying mechanisms of inhibitory control impairments.

Nevertheless, there is another theoretical viewpoint which does not appeal inhibitory control that could help to explain the semantic plausibility effect. Potter [79] argued that recall of sentences that deviate from conceptual representations stored in LTM (such as implausible sentences) are processed and regenerated by a discrete conceptual STM component that is not a core part of LTM but interacts with LTM. On the basis of this view, the partial semantic plausibility effect evident in the preparation time results could reflect retrieval from the hypothesized conceptual STM component, rather than the effect of inhibitory control, or the need for inhibitory control may be located in the comparison of the “novel” (i.e., implausible conceptual representation) with other conceptual representations stored in LTM. This possible explanation has not been discussed explicitly before in the sentence recall literature, neither in aphasia nor in neurotypical individuals. Given the interactive nature of verbal STM and LTM and related debates about the precise architecture of this relationship in terms of processing and control components [65,73,92], future research with more robust methodology than the present study should explore both explanations (inhibitory control, conceptual STM).

## 5. Conclusions

Despite the absence of interactions between aphasia groups and sentence types, which, had they been evident, would have enabled us to gain a deeper understanding about the nature of the verbal abilities in latent and anomic aphasia, this paper brought to the fore the need for further studies in these milder forms of aphasia, which have seldom been examined at sentence level. It appears that processing speed as indexed by temporal speech measures is differentially compromised between people with latent and anomic aphasia and crucially, as other authors [12,27] noted, between people with latent aphasia and controls. We also advanced an explanation based on the conceptual STM component as to why semantically implausible sentences are typically more erroneous and as we have shown possibly also slower in recall.

## Figures and Tables

**Table 1:** Demographic information.

	Latent	Anomic	Controls
Characteristics			
age	59.5 (14.3)	60.1 (12.5)	61.2 (13.6)
education	15.5 (2.7)	14.9 (2.6)	15.6 (3.3)
sex	F = 12; M = 5	F = 8; M = 22	F = 9; M = 4
time post onset	4.7 (*3.7*)	4.6 (*3.8*)	n.a.
Aphasia Quotient	96.3 (*1.8*)	85.3 (*7.8*)	n.a.
word span	3.8 (*0.9*)	3.1 (*1.1*)	4.9 (*0.3*)

Note: n.a. = not applicable.

**Table 2:** Descriptive statistics (accuracy, temporal speech measures).

		Latent	Anomic	Controls
Measures	Sentence Types			
accuracy	Plausible simple	11.71 (*1.24*)	11.31 (*1.76*)	12.00 (*0.00*)
	Implausible simple	11.56 (*0.75*)	10.57 (*2.46*)	12.00 (*0.00*)
	Plausible complex	11.03 (*1.99*)	9.75 (*2.52*)	11.92 (*0.27*)
	Implausible complex	11.41 (*1.40*)	10.00 (*2.47*)	11.69 (*1.05*)
response	Plausible simple	3658 (*1401*)	4569 (*3006*)	2998 (*890*)
	Implausible simple	4608 (*2019*)	6338 (*4985*)	2853 (*817*)
	Plausible complex	4342 (*2309*)	5844 (*4667*)	2894 (*551*)
	Implausible complex	5397 (*3256*)	5971 (*3959*)	3353 (*1113*)
preparation	Plausible simple	779 (*558*)	761 (*295*)	696 (*270*)
	Implausible simple	1306 (*838*)	1684 (*1994*)	619 (*355*)
	Plausible complex	764 (*804*)	931 (*727*)	546 (*207*)
	Implausible complex	1243 (*1706*)	876 (*536*)	655 (*412*)
speech	Plausible simple	2467 (*662*)	2857 (*1252*)	2257 (*617*)
	Implausible simple	2620 (*857*)	3012 (*1291*)	2234 (*582*)
	Plausible complex	2842 (*893*)	3280 (*1701*)	2340 (*453*)
	Implausible complex	3032 (*1188*)	3244 (*1430*)	2606 (*848*)
pause	Plausible simple	412 (*678*)	951 (*2019*)	44 (*158*)
	Implausible simple	682 (*1082*)	1611 (*0*)	0 (*2431*)
	Plausible complex	736 (*1182*)	1609 (*3379*)	8 (*41*)
	Implausible complex	1122 (*1600*)	1859 (*2599*)	91 (*286*)

**Figure A1:**
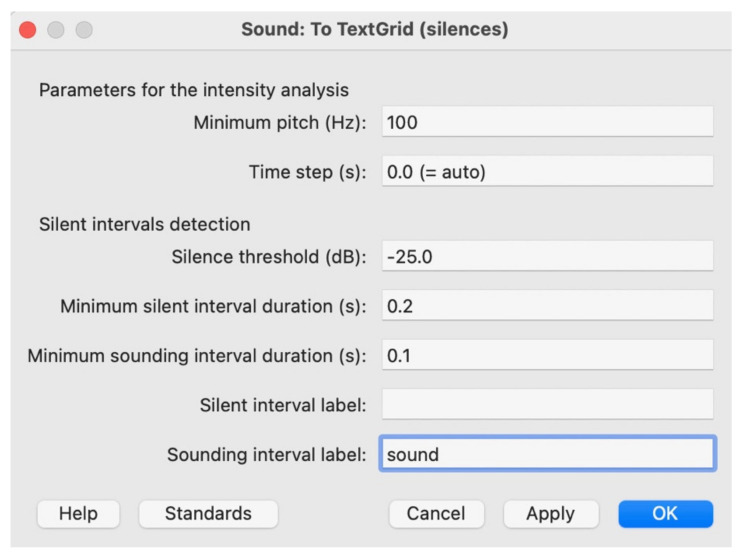
The Praat settings for the semi-automated sound-pause analysis.

**Figure A2:**
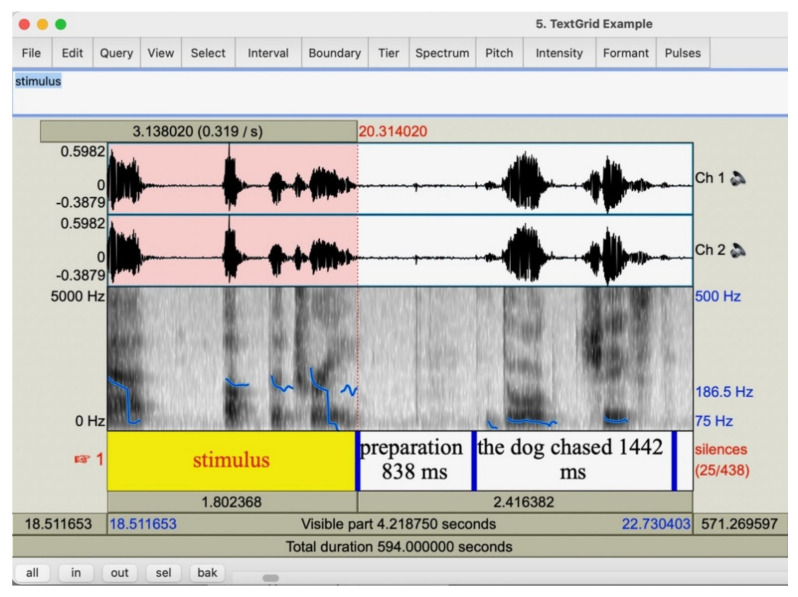
An example of speech segmentation.

## Data Availability

The data are available for the first author and also through Brain Sciences.

## Appendix A

**Table A1:** AphasiaBank participant codes.

Anomic Aphasia	Latent Aphasia
Adler17a	Acwt04a
Adler24a	Adler03a
Bu04a	Fridriksson07a
Bu06a	Fridriksson11a
Bu12a	Kurland04a
Elman05a	Kurland06a
Elman07a	Scale16a
Elman10a	Scale20a
Elman15a	Scale21a
Fridriksson04a	Tcu09a
Kansas18a	Tcu10a
Kansas19a	Tucson06b
Kurland07a	Tucson18a
Kurland08a	Whiteside17a
Kurland10a	Williamson05a
Scale17a	Williamson13a
Scale30a	Wozniak06a
Tap08a	
Tcu05a	
Whiteside13a	
Williamson08a	
Williamson09a	
Williamson17a	
Williamson18a	
Williamson24a	
Wozniak01a	
Wright202a	

## Appendix B

**Table A2:** Sentence recall stimuli.

	Semantics	Syntax
Sentences (and Order of Presentation)		
The dog chased the cat up the tree	plausible	simple
The bird was caught by the worm	implausible	complex
Books like to read children	implausible	simple
Ice cream tastes good in the summer	plausible	simple
Beautiful flowers smelled the lovely women	implausible	simple
The man saw the boy that the dog chased	plausible	complex
The tiger was clawed by the lion	plausible	complex
Bad weather was caused by long airplane delays	implausible	complex
